# Endobronchial Fluoroscopy-Guided Management of Persistent Air Leak After Video-Assisted Thoracoscopic Surgery (VATS)-Assisted Deroofing of Pleural Hydatid Cysts

**DOI:** 10.7759/cureus.106805

**Published:** 2026-04-10

**Authors:** Amol Sood, Jyoti Sharma, Yashwant S Rathore, Rajendra K Behera, Ashu S Bhalla

**Affiliations:** 1 Department of Surgical Disciplines, All India Institute of Medical Sciences, New Delhi, New Delhi, IND; 2 Department of Radiodiagnosis and Interventional Radiology, All India Institute of Medical Sciences, New Delhi, New Delhi, IND

**Keywords:** bronchoscopic coil embolisation, hydatid cyst deroofing, persistent air leak (pal), pulmonary hydatid disease, video-assisted thoracoscopic surgery (vats)

## Abstract

Persistent air leak (PAL) is a challenging postoperative complication after thoracic surgery. We present the case of a woman in her 30s with hydatid disease involving the abdomen and lungs, who developed PAL following video-assisted thoracoscopic surgery (VATS)-assisted deroofing of left pleural hydatid cysts. Conservative management with chest tube drainage and negative suction failed to resolve the air leak. On postoperative day seven, bronchoscopy with balloon occlusion localized the leak to the upper lobe of the left lung. A combined bronchoscopy and fluoroscopy-guided approach was used for coil embolization and glue placement in the involved airways, successfully sealing the alveolo-pleural fistula. The patient showed complete resolution of the air leak and was discharged in stable condition after chest tube removal. This case highlights the role of bronchoscopy-guided interventions as a minimally invasive alternative for PAL management, particularly in peripheral airway leaks, reducing the need for surgical re-intervention.

## Introduction

Persistent air leak (PAL) is a challenging postoperative condition, defined as the continued escape of air from the lung parenchyma into the pleural cavity for more than five to seven days [1].

It is most commonly encountered following thoracic surgical procedures and may lead to prolonged chest tube drainage, extended hospital stay, and increased patient morbidity [2]. Although many air leaks resolve with conservative measures, such as chest drainage and negative suction, persistent cases often require additional intervention. In recent years, several bronchoscopic techniques, including endobronchial valves, coils, and sealants, have been described as minimally invasive options for the management of PAL [2,3]. The liver is generally the most commonly affected organ by hydatid disease. Pleural involvement generally occurs secondary to rupture of a hepatic or pulmonary hydatid cyst inside the pleural cavity [4]. We describe the case of a patient who developed PAL following video-assisted thoracoscopic surgery (VATS)-assisted deroofing of left pleural hydatid cysts and was successfully managed using a bronchoscopic and fluoroscopy-guided approach.

## Case presentation

A woman in her 30s, a known case of lung and abdominal cystic hydatid disease, presented to our hospital with the complaint of left-sided chest discomfort for three months. She did not have any other complaints. She had a previous history of splenectomy 15 years back for splenic cystic hydatid disease. She had also been diagnosed with a left lung hydatid cyst with pleural effusion secondary to rupture of the lung hydatid eight years back, which was managed conservatively with chest tube placement and medications. At the current presentation, the patient had an unremarkable general physical and respiratory system examination. Her vital parameters, including heart rate, blood pressure, respiratory rate, oxygen saturation, and temperature, were all within the normal range. Her total leukocyte count was 8,830/mm^3^ (normal range: 4,000-11,000/mm^3^).

**Figure 1:**
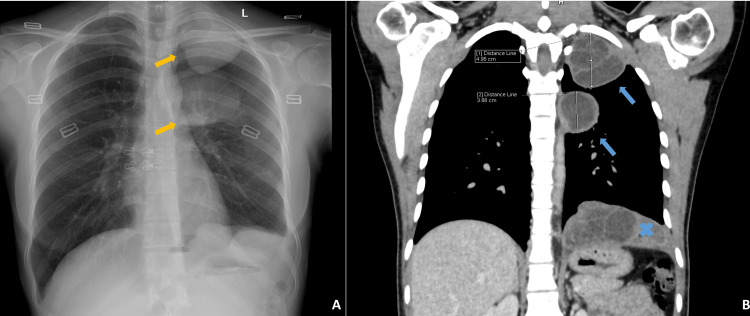
Preoperative imaging findings (A) Chest radiograph showing two well-defined, rounded masses in the upper and middle zones of the left hemithorax (yellow arrows). (B) Contrast-enhanced CT chest (coronal section) demonstrating two well-defined, multilocular, pleural-based, cystic lesions with internal septations (blue arrows), corresponding to the radiographic findings. A multiloculated cystic lesion is also noted in the splenic bed (blue cross).

A chest radiograph was done, which showed well-defined, rounded masses in the middle and upper zones of the left thoracic cavity (Figure 1). A contrast-enhanced computed tomography (CECT) of the lung showed two well-defined multilocular cysts showing internal septations along the superior segment of the lower lobe of the left lung and the apico-posterior segment of the upper lobe of the left lung, respectively. The cysts did not exhibit an obvious communication with the airways (Figure 1). The main differential diagnoses considered at this point were pleural hydatid cyst, empyema thoracis, and mesothelial cyst. Based on the imaging characteristics and previous history of the patient, and given that the patient was afebrile with normal laboratory parameters, a diagnosis of left pleural cystic hydatidosis was made.

The patient was planned for VATS-assisted deroofing of the left pleural hydatid cysts. The procedure was done under general anesthesia. The patient was placed in the right lateral position with the operating table flexed at the level of the lower chest, and single lung (right-sided) ventilation was done using a 35 French double lumen endotracheal tube. Intraoperatively, dense adhesions were encountered between the left visceral and parietal pleura. The two cystic lesions identified on imaging were localised and were confirmed to be hydatid cysts containing multiple daughter cysts within. Deroofing of both cysts with evacuation of contents was done, taking care to prevent spillage of cyst contents. Additionally, 10% povidone-iodine was instilled into the cyst cavities after evacuation and left inside for 20 minutes to ensure sterilization of the cyst cavity. Scolicidal agents, such as 10% povidone-iodine, 3% hydrogen peroxide, 95% ethyl alcohol, and 20% saline, are commonly used for instillation into the hydatid cyst, and the surrounding operative area is protected using sponges impregnated with these agents, both with the aim of preventing intraperitoneal spread of the parasite and preventing local tissue toxicity [5].

An air leak test was performed at the end of the procedure by instilling normal saline into the left pleural cavity, inflating the lung, and observing for bubble formation in the instilled saline, which would be indicative of an air leak. This test was negative for air leak. A 32-French chest tube was placed in the left pleural cavity at the end of the procedure.

The patient was shifted to the postanesthesia care unit for monitoring. Intravenous analgesics were given around the clock to ensure adequate pain relief for the patient.

**Figure 2:**
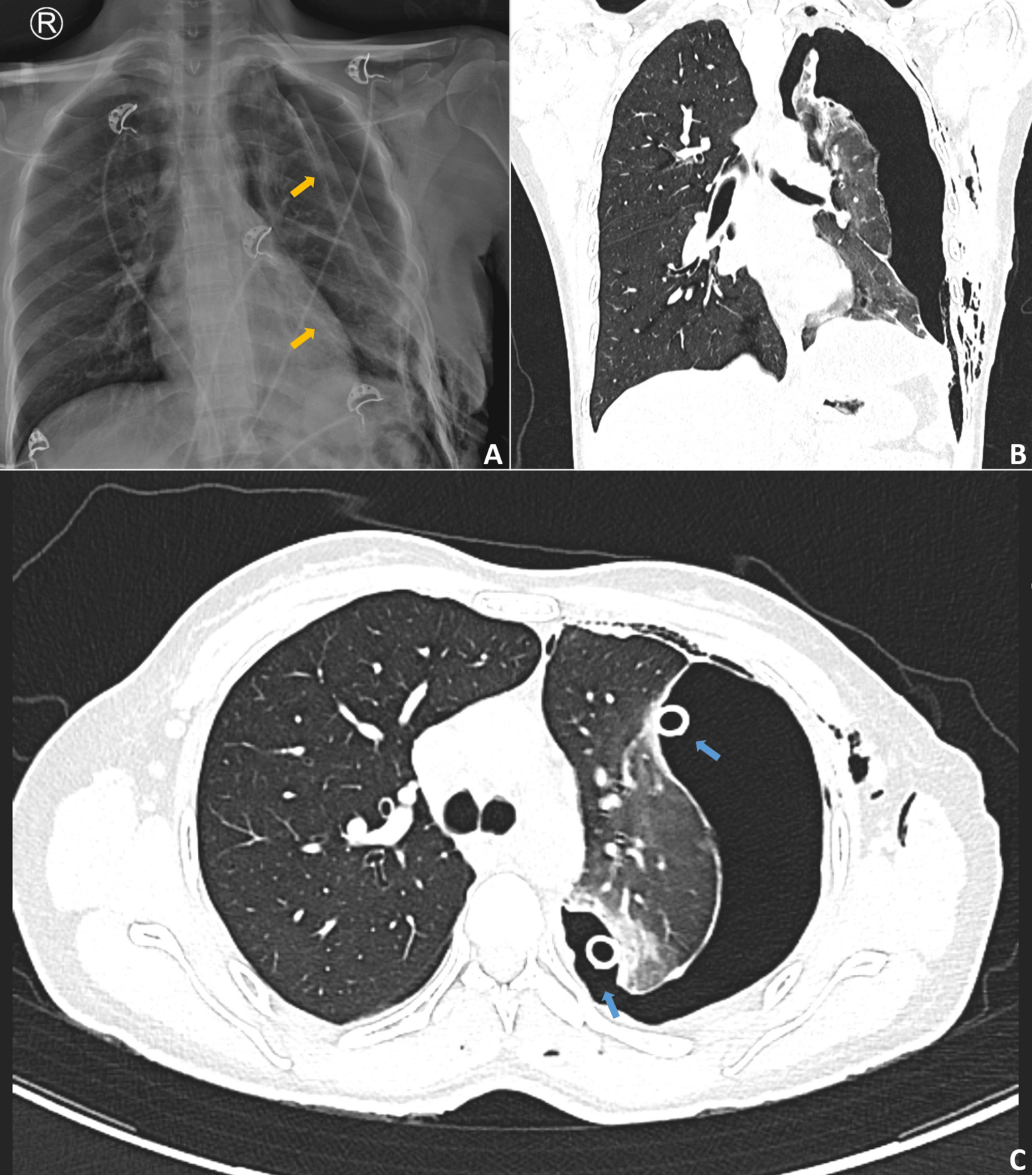
Postoperative imaging findings (A) Chest radiograph obtained six hours after VATS-assisted deroofing of left pleural hydatid cysts, showing a left-sided pneumothorax with two chest tubes in situ (yellow arrows). (B, C) Contrast-enhanced CT chest (postoperative day one) demonstrating a large left-sided pneumothorax with compression of the left lung parenchyma and two chest tubes in the left pleural cavity (blue arrows).

Six hours postoperatively, grade 2 air leak [6] was noted from the chest tube, and a chest radiograph showed a left pneumothorax (Figure 2). Since the patient did not have any dyspnea and was hemodynamically stable, a second chest tube was placed in the left pleural cavity, and conservative measures for air leak management were initiated, which included applying negative suction (at a pressure setting of -10 mmHg) to one of the chest tubes, active chest physiotherapy, and antibiotics. In many cases, conservative measures, including pleural drainage, irrigation, appropriate antibiotics, and nutritional support, have been shown to be effective in achieving fistula closure and, hence, the rationale for approaching the case conservatively in the initial few days of air leak [7]. A CECT chest done on postoperative day one showed a large left-sided pneumothorax with collapsed left lung parenchyma. However, no air leak (fistulous communication between the bronchus and the pleura) was evident on the CT (Figure 2). There was a persistent grade 2 air leak from both chest tubes. Since the patient remained asymptomatic and hemodynamically stable, maintaining oxygen saturation of >95% on room air, conservative management of the air leak was continued till postoperative day seven. In addition, since many air leaks have been shown to resolve spontaneously with conservative measures in the initial five to seven days, it was considered to defer intervention till the seventh postoperative day [1]. 

**Figure 3:**
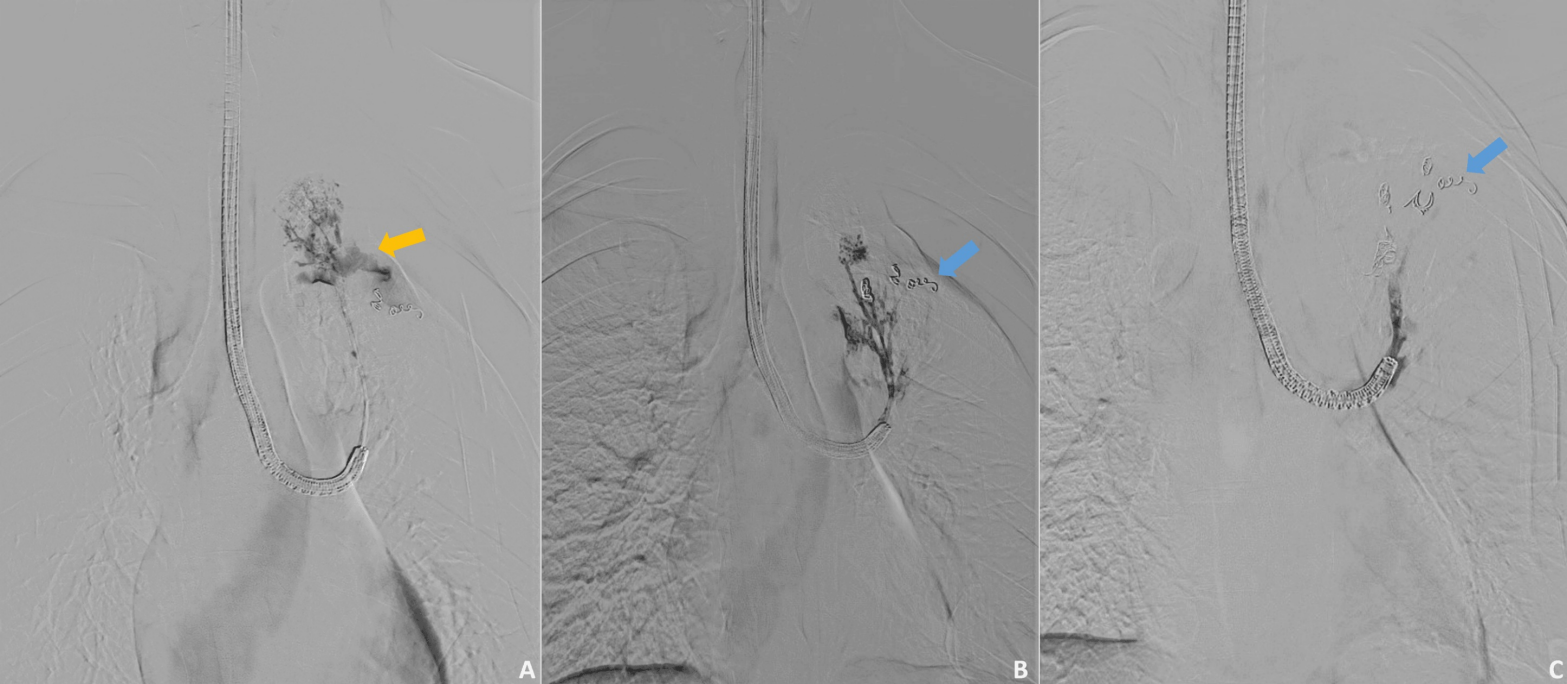
Digital subtraction bronchography findings (A) Digital subtraction bronchogram of the left upper lobe showing contrast leak from subsegmental bronchi supplying the posterior segment (yellow arrow). (B, C) Post-embolization bronchograms demonstrating normal opacification of the tracheobronchial tree with no contrast leak. Radio-opaque vascular embolization coils are seen within the treated bronchi (blue arrows).

Due to a non-resolving grade 2 air leak from the chest tubes and a persistent pneumothorax on serial chest radiographs, bronchoscopy was done under local anesthesia on postoperative day seven. Serial balloon occlusion of the left lung segmental bronchi showed complete resolution in air leak on occlusion of the left lung upper lobe apical and posterior segments, thus localizing the leak to these segments. Since the peripheral airways were difficult to approach via bronchoscopy alone, to manage the PAL, the patient was planned for a combined bronchoscopy and fluoroscopy approach, guided coil embolization, and glue placement in the bronchi supplying the affected lung segments. The procedure was done under local anesthesia on postoperative day 10. Multiple air leaks were noted from several subsegmental bronchi supplying the posterior and apical segments of the upper lobe of the left lung. The involved subsegmental bronchi were selectively embolized with vascular embolization coils (0.018", 3 mm x 5 cm vascular embolization coil by MicroNester®, Cook Medical, Bloomington, IN). Multiple coils were placed in each subsegmental bronchus, and the residual gap between the coils was plugged with approximately 1 mL of cyanoacrylate glue. The bronchogram post-procedure did not show any air leak from the involved segments (Figure 3). The patient tolerated the procedure well.

**Figure 4:**
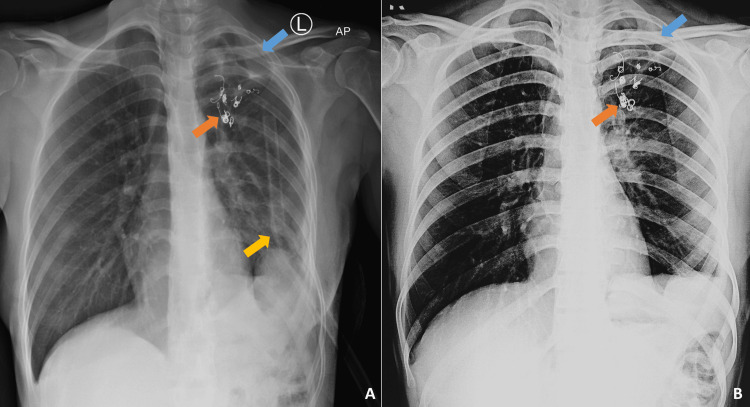
Post-embolization and follow-up imaging (A) Chest radiograph obtained two days after coil embolization and glue placement, showing near-complete expansion of the left lung with a small residual left pneumothorax (blue arrow). One chest tube has been removed, with the remaining tube in situ (yellow arrow). (B) Follow-up chest radiograph obtained 14 days after discharge demonstrating stable findings with near-complete left lung expansion and a small residual pneumothorax (blue arrow). Radio-opaque vascular embolization coils are visible in both panels (orange arrows).

To ensure adequate lung expansion post procedure, one of the chest tubes was kept on negative suction overnight (at a pressure setting of -10 mmHg). On the day following the procedure, there was complete resolution of air leak from both chest tubes, and the chest radiograph showed near complete expansion of the left lung (Figure 4). Both chest tubes were removed sequentially over the subsequent three days, after ensuring the absence of air leak and stable chest radiographs. The patient was discharged on postoperative day 17 in stable condition. The patient was called for follow-up 14 days after discharge. She continues to do well and has resumed all daily activities. Chest radiograph obtained at the follow-up visit showed stable findings with near complete expansion of the left lung (Figure 4). The patient has been advised to continue taking tablet albendazole 400 mg twice daily for at least six months and has been called for follow-up after three months.

## Discussion

Echinococcosis, or hydatid disease, is caused by infection with the *Echinococcus *tapeworm. Of its species, *Echinococcus granulosus* produces cystic echinococcosis, while *E. multilocularis *leads to alveolar echinococcosis [8]. Cystic echinococcosis usually affects the liver and lungs. Pleural disease is rare and generally secondary to rupture of pulmonary hydatid cysts [9,10]. In this case, the patient had hydatid disease involving both the abdomen and thoracic cavity, with pleural disease due to rupture of a pulmonary hydatid cyst eight years earlier.

PAL is defined as continuous leakage of air into the pleural cavity for more than five to seven days [1], or >15-20 mL/min for over five days on digital drainage systems [3]. It complicates 26% of lobectomies and up to 46% of lung volume reduction surgeries [3]. Other causes include spontaneous pneumothorax, necrotising pneumonia, mechanical ventilation-related barotrauma, and thoracic trauma [3]. Risk factors include major resections, female sex, reduced forced expiratory volume in 1 second (FEV1) or diffusing capacity of the lungs for carbon monoxide (DLCO), incomplete fissures, pleural adhesions, chronic obstructive pulmonary disease (COPD), diabetes, steroid use, and smoking [3,11]. PAL predisposes to atelectasis, pneumonia, empyema, delayed mobilisation, prolonged hospitalization, higher costs, and increased morbidity [12]. In our patient, PAL developed postoperatively despite the absence of clear preoperative airway communication on imaging. This could be due to the adhesiolysis of the dense adhesions encountered intraoperatively between the visceral and parietal pleura, which could have led to a breach in the visceral pleura and hence exposed the airways to the pleural cavity, leading to a PAL.

Air leaks are graded by Cerfolio [6]: grade 1 (on forceful expiration), grade 2 (on normal expiration), grade 3 (on inspiration), and grade 4 (continuous throughout respiration).

Conservative treatment includes intercostal drainage with or without suction, chest physiotherapy, and antibiotics. Larger or persistent leaks often require intervention [13]. The American College of Chest Physicians' 2001 consensus statement on spontaneous pneumothorax provides brief guidance on managing PAL. It suggests that, if an air leak continues beyond four days, a thoracic surgical opinion should be sought. For patients who are not suitable candidates for surgery, chemical pleurodesis is recommended as an alternative approach [14]. The latest British Thoracic Society pleural disease guidelines on spontaneous pneumothorax also touch upon the management of PAL. They advise seeking a surgical opinion if the air leak continues beyond 48 hours. For patients who are not fit for surgery, options such as chemical pleurodesis or the use of a chest tube connected to a one-way flutter valve are suggested as alternative approaches [15]. While surgery was once the standard, less invasive bronchoscopy-guided methods are now common, including endobronchial or intrabronchial valves (EBV/IBV), spigots, adhesives, vascular coils, and septal occluder devices [2,3].

Endobronchial closure was first described in 1977, when Harmann et al. and Ratliff et al. reported success using tissue glue and lead shot, respectively [16,17]. Later, Ponn et al. achieved closure of peripheral air leaks in five patients using coils [18]. Watanabe et al. reported success in four of five patients using coils plus cyanoacrylate or fibrin glue [19].

Katoch et al. treated 25 chronic air leaks with coils, glue, or both; although initial success was seen, many required repeat interventions, and 18 ultimately needed surgery [20]. Marwah et al. managed 11 postoperative air leaks, with closure in nine (81.8%) patients [21]. Bai et al. reported success in five of six cases, with failure in a large air leak due to a 5.8 mm broncho-pleural fistula, suggesting efficacy mainly for defects <2 mm [22].

These reports support coils with glue as an effective option for small, peripheral air leaks, consistent with our case where an alveolo-pleural fistula was sealed. Notably, unlike prior cases of PAL after lung resection for tuberculosis or hydatid disease, our patient developed PAL after deroofing of a pleural hydatid cyst, which is a rare scenario.

## Conclusions

Persistent air leak following thoracic surgery can be challenging for both the patient and the treating team, particularly when conservative measures fail. Timely localization of the leak is critical in guiding further management. This case illustrates that selective bronchoscopic coil embolization with adjunctive glue placement, performed under fluoroscopic guidance, can effectively manage peripheral air leaks. In carefully selected patients, such an approach may help avoid surgical re-exploration and facilitate earlier recovery. There are numerous medical and surgical options in the treatment of PAL. The clinical variability of PAL from patient to patient; the lack of guidelines, consensus, or algorithms for the treatment of PAL; and the absence of strong evidence supporting the use of any one particular device or technique suggest that the treatment of PAL should be individualized to each patient.
